# Particle partitioning and geography drive divergent microbial assembly and network connectivity in coastal South China Sea

**DOI:** 10.3389/fmicb.2025.1738577

**Published:** 2026-01-23

**Authors:** Shimei Pang, Songze Chen, Ziqiu Lin, Wei Xie, Yongqian Xu, Chuanlun Zhang

**Affiliations:** 1 Shenzhen Key Laboratory of Marine Archaea Geo-Omics, Department of Ocean Science and Engineering, Southern University of Science and Technology, Shenzhen, China; 2 Shenzhen Ecological and Environmental Monitoring Center of Guangdong Province, Shenzhen, China; 3 Department of Ocean Science, The Hong Kong University of Science and Technology, Sai Kung, Hong Kong SAR, China; 4 School of Marine Sciences, Sun Yat-sen University &Southern Marine Science and Engineering Guangdong Laboratory (Zhuhai), Zhuhai, China; 5 Research Center for Ocean Negative Carbon Emissions, Xiamen, China

**Keywords:** free-living, microbial community assembly, network analysis, particle-attached, South China Sea

## Abstract

A pronounced nutrient gradient spans from the eutrophic Pearl River Estuary (PRE) to the oligotrophic Northern South China Sea (NSCS), yet its influence on microbial community distribution and cross-domain interactions remains poorly understood. Here, we combined rRNA amplicon sequencing, cross-domain network analysis, and null model approaches to characterize and compare the community structure, assembly processes, and interactions of archaeal, bacterial, and eukaryotic communities in particle-attached (PA) and free-living (FL) fractions along the PRE-NSCS gradient. In the PRE, microbial community assembly was predominantly governed by stochastic processes, resulting in pronounced differences in potential connectivity predicted by null models. Notably, ammonia-oxidizing archaea associated with particles likely functioned as key connectors linking nitrification modules with heterotrophic clusters. In contrast, in the NSCS, cross-domain network analysis revealed that eukaryotes play a central role in maintaining inter-domain connectivity, while FL heterotrophic bacteria formed tightly coupled core networks with their autotrophic partners. Consistent with these patterns, validated topological structures indicated that PRE communities are dominated by stochastic processes (dispersal limitation and drift), whereas NSCS FL communities are primarily shaped by homogeneous selection. Collectively, these results demonstrate that geography and particle partitioning jointly regulate microbial community assembly and network connectivity, thereby influencing distinct microbial remineralization pathways associated with particulate versus dissolved organic matter, and providing new insights into carbon-nitrogen coupling in dynamic coastal ecosystems.

## 1 Introduction

Marine microorganisms contribute roughly half of the global primary production and serve as fundamental drivers of Earth’s biogeochemical cycles (Ducklow et al., 1986; Field et al., 1998; Azam and Malfatti, 2007; Sunagawa et al., 2015; Jiao et al., 2024). They maintain a relatively stable global ecosystem through their extensive metabolic diversity collectively, such as photosynthesis, heterotrophic respiration, and diverse redox reactions (Arrigo, 2005; Jiao et al., 2010; Lam and Kuypers, 2011). They exist as either free-living (FL) or particle-associated (PA) forms, and these two forms are the key factors influencing the ecological functions of these microorganisms (Mestre et al., 2018; Li et al., 2021a; Rosenberg et al., 2021). Genomic evidence suggested that microbial communities in PA exhibit a greater metabolic diversity and an enhanced capacity for degrading organic particles (Leu et al., 2022). Additionally, the heterogeneity of the particle microenvironment significantly influences their metabolic strategies and community interactions (Suter et al., 2018; Yeh and Fuhrman, 2022). It is important to understand how these lifestyle forms respond to environmental gradients in dynamic coastal ecosystems.

Coastal ecosystems, which include estuaries and marginal seas, function as dynamic interfaces where terrestrial and marine systems intersect (Barceló et al., 2023). The diverse geomorphology of these areas combined with intense water mixing and rapid changes in physical and chemical conditions produces environments that are both highly productive and extremely diverse. These environments function as hotspots for biogeochemical processes which control worldwide carbon and nutrient distribution (Cloern et al., 2017; Carstensen et al., 2018; Chen et al., 2025). A notable example is the Pearl River Estuary (PRE), which establishes a significant salinity gradient that functions as a primary ecological filter (Wong et al., 2004; Zhou et al., 2011; Xu et al., 2018; Fan et al., 2023). This gradient rapidly restructures and functionally modifies microbial assemblages, with salinity and temperature serving as the main deterministic factors (Mo et al., 2021; Wu et al., 2024; Zhang et al., 2024). In contrast, the oligotrophic northern South China Sea (NSCS) faces different selection pressures, where the distribution patterns and network connectivity of microorganisms are primarily determined by the vertical distribution of particle size (Zheng et al., 2021; Ma et al., 2024; Zhang et al., 2025). This phenomenon further influences carbon export efficiency (Li et al., 2018). The transition from nutrient-rich PRE waters to oligotrophic NSCS provides a significant and ideal model system for studying how geographical factors shape microbial assembly and community structure (Chen et al., 2020; Li et al., 2021b; Wu et al., 2023). Thus, the PRE-NSCS continuum provides an ideal natural model to investigate how environmental gradients and lifestyle strategies jointly shape microbial assembly and function.

Recent studies have increasingly emphasized the joint consideration of bacteria, archaea, and eukaryotes to capture cross-domain ecological interactions (Moissl-Eichinger et al., 2018; Weiland-Bräuer, 2021). Within a community, if only bacteria are present, the community may lack functional complementarity or recovery pathways after disturbance. However, if archaeal or eukaryotic members are involved, it may enhance functional recovery capacity (Gallardo-Navarro et al., 2024). Furthermore, Earth’s element cycles often involve the concerted action of multi-domain microorganisms, thus making cross-domain collaboration increasingly important from a functional ecology perspective (Rowan-Nash et al., 2019; Dong et al., 2022; Zhang et al., 2023; Pereira et al., 2024). Such interactions often enhance community functional resilience and contribute to key biogeochemical processes. For instance, previous studies have revealed that ammonia-oxidizing archaea (AOA; Marine Group I) interact with ammonia-oxidizing bacteria during nitrification, together regulating key steps of the marine nitrogen cycle (He et al., 2018; French et al., 2021). In contrast, archaea Marine Group II (MGII) exhibit a synergistic relationship with phytoplankton in the carbon cycle (Xie et al., 2017; Jain and Krishnan, 2021; Chen et al., 2022). Therefore, we hypothesize that in the PRE to NSCS gradient system, the three domains of life, bacteria, archaea, and eukaryotes, exhibit domain-specific assembly patterns and ecological strategies, either for FL or PA states (Li et al., 2023b; Ramond et al., 2023; Hou et al., 2024).

The assembly of the complex communities is governed by a balance between deterministic and stochastic processes (Aguilar and Sommaruga, 2020; Riddley et al., 2025). Changes in environmental conditions, particle aggregation patterns, and spatial scales alter the relative importance of different processes in community formation (Trego et al., 2021; Lin et al., 2023; Bontemps et al., 2024). In marine and coastal systems, bacterial communities are often more strongly influenced by homogeneous selection, whereas eukaryotic communities tend to exhibit higher stochasticity, and archaeal communities may rely on more specialized survival strategies, depending on environmental conditions and spatial scales (Trego et al., 2021; Qiao et al., 2024; Dąbrowska et al., 2025; Riddley et al., 2025). However, systematic comparative studies across these three domains under the same environmental gradient are relatively scarce, largely due to challenges in cross-domain standardized molecular analytical methodologies.

In addition to the assembly process, ecological network analysis provides insights into interactions, core species, modular structure, network connectivity, and robustness at the community structure level, thereby helping to assess the community’s response capacity to environmental disturbances (Oña and Kost, 2022; Kajihara and Hynson, 2024). These network metrics also serve as key indicators for understanding community characteristics and ecological stability (Olesen et al., 2007; Grilli et al., 2016; Meghanathan, 2021). Archaea and eukaryotes attached with particles constitute key groups that can drive network connections and significantly enhance ecosystem resilience (Jain and Krishnan, 2021; Guo et al., 2022; Li et al., 2023a). Furthermore, stochastic processes can facilitate the formation of bridging groups, thereby effectively enhancing cross-domain connectivity and playing a positive role in stabilizing communities (Zhou and Ning, 2017; Ayala-Ortiz et al., 2025). Environmental filtering and diffusion limitation often exacerbate spatial heterogeneity and modularity, thereby weakening cross-domain interactions. However, moderate randomness, referring to situations in which stochastic processes coexist with deterministic environmental filtering, has been shown to enhance network connectivity by preventing excessive modular isolation (Huber et al., 2020; Yang et al., 2022; Li et al., 2023c). Therefore, integrating network metrics with environmental factors and assembly processes is essential for explaining microbial community stability and function.

Therefore, to synthesize these concepts, this study investigates the assembly strategies and cross-domain interactions of the three domains of life across the PRE-NSCS continuum. This study analyzed two types of samples, FL and PA, using high-throughput amplicon sequencing technology. Combined with null model analysis and ecological network construction, it focuses on exploring three core questions:

1. What differences exist in community composition, core taxa, and assembly processes among the three major biomes within a significant ecological gradient?
2. Are there consistent or differential characteristics in FL and PA communities during intra-domain and cross-domain assembly processes?
3. How are these differences associated with network connectivity?

## 2 Materials and methods

### 2.1 Sample collection and physicochemical measurements

The survey covered 16 stations with different depths in the PRE and NSCS, producing 52 samples of FL and PA microbial communities (Supplementary Figure S1; Supplementary Table 1). Sampling stations were categorized into the Pearl River Estuary (PRE) and the northern South China Sea (NSCS) based on natural bathymetric differences, with PRE stations located in shallow estuarine waters and NSCS stations located in deeper offshore waters. Locations in the PRE were marked with ‘P’ and those in the NSCS with ‘S’. A CTD system (SBE 9–11 Plus, Sea-Bird Electronics, USA) with 12 L Niskin bottles was used to measure hydrographic parameters such as depth, temperature, and salinity. The research included samples taken from various depth layers, with depths ranging from 5 to 25 m in the PRE and 5 to 67 m in the NSCS. Detailed related site data can be referred to the previous article of our team (Chen et al., 2022).

Seawater ranging from 2 to 4 liters was filtered in sequence using 2.7 μm glass microfiber membranes followed by 0.22 μm cellulose nitrate membranes to distinguish PA from FL fractions. Particles and their communities that are trapped by 2.7 μm prefilters are identified as the PA community in this study, while those that pass through these filters but are stopped by 0.22 μm filters are identified as FL communities (Cui et al., 2020). Therefore, FL eukaryotes mainly consist of small eukaryotes that are not bound to large particles and their FL life stages. The filter membrane was quickly frozen using liquid nitrogen within 20 min for DNA extraction, and the filtrate was kept at −20 °C for nutritional analysis. Ammonium levels were measured using the indophenol blue spectrophotometric technique (Sims et al., 1995). A continuous flow autoanalyzer was employed to measure nitrate, nitrite, phosphate, and silicate (Technicon AA3, Germany). All procedures were conducted in accordance with institutional and national guidelines, no endangered species were involved.

### 2.2 DNA extraction and amplicon sequencing

With slight modifications, DNA was extracted from filters using the FastDNA SPIN Kit for Soil (MP Biomedicals, USA) (Tournier et al., 2015). The steps included cryopreserving the filters, lysing them, precipitating proteins, and purifying through Spin columns. Using the Illumina MiSeq platform, amplicon sequencing was conducted targeting the universal 16S/18S rRNA genes with the primer pair 515F/926R (5′-GTGYCAGCMGCCGCGGTAA-3′)/(5′-CCGYCAATTYMTTTRAGTTT-3′) (Walters et al., 2015). Cutadapt was used to trim low-quality sequences (average quality < 20) and remove primer regions (Martin, 2011). The DADA2 plugin was used to truncate, denoise, and remove chimeras from the trimmed sequences. Taxonomic classification of ASVs was performed using the SILVA 138.1 database with the classify-sklearn plugin in QIIME2 (Bolyen et al., 2018; Quast et al., 2013).

The universal primer pair 515F/926R amplifies small subunit (SSU) rRNA genes from bacteria, archaea, and eukaryotes. After quality control and ASV inference, taxonomic assignment was performed against the SILVA SSU rRNA database. ASVs classified as Eukaryotes were extracted for downstream analyses. To ensure the reliability of eukaryotic community profiles, only ASVs with confident taxonomic assignments (bootstrap ≥70%) were retained, while non-eukaryotic, ambiguous, or low-abundance ASVs occurring in fewer than 2 samples were removed. Previous evaluations have demonstrated that recovery of 18S rRNA sequences from 515F/926R amplicons depends primarily on downstream bioinformatic filtering rather than primer bias itself (Parada et al., 2016). Accordingly, although differential amplification efficiencies across domains, particularly for eukaryotic taxa, cannot be entirely excluded, primer bias is unlikely to be the primary source of systematic variation in the resulting community profiles (Parada et al., 2016). Furthermore, this primer pair offers high coverage and minimal bias in marine systems, ensuring robust comparability across domains (McNichol et al., 2021). Serial filtration protocols minimized size-related artifacts in community assembly and network inferences (Levine et al., 2024).

To ensure uniform sequencing depth for subsequent statistical and ecological analyses, ASV tables were rarefied in R using the rrarefy function in the vegan package. Rarefaction was performed separately for each domain by randomly subsampling each sample to the minimum sequencing depth within that dataset: 668 reads per sample for archaea, 77,367 reads per sample for bacteria, and 2,598 reads per sample for eukaryotes.

### 2.3 Data processing and statistical analyses

The sequence data were standardized to a uniform sequencing depth across all samples. We created the sampling map using Ocean Data View.^1^ Community assembly was quantified using the iCAMP phylogenetic-bin-based null model with 1,000 randomizations, using standard thresholds for *β*NRI (±1.96) and RCbray (±0.95) to assign ecological processes (Ning et al., 2020). To better interpret and present data, Chiplot created various visualizations, including stacked bar charts, non-metric multidimensional scaling (NMDS), canonical correspondence analysis (CCA), phylogenetic trees, and heatmaps (Li et al., 2023a). Prior to CCA, environmental variables (depth, temperature, salinity, and nutrient concentrations) were examined for multicollinearity using variance inflation factors (VIFs), and no severe collinearity was detected (all VIF < 10). Therefore, all variables were retained in the final CCA model. In *R* (vegan v2.6–4), variance partitioning analysis (VPA) was utilized to pinpoint the primary environmental influences (Lyu et al., 2023). Cross-domain interaction networks were constructed in Wekemo Bioincloud, retaining the top 1% abundant genera and filtering edges by Pearson correlation (|*r*| > 0.8, FDR-adjusted *p* < 0.05), with isolated nodes removed (Gao et al., 2024). The core taxa were defined as those with a relative abundance >0.5% in at least one sample, and keystone roles were assigned based on Zi and Pi thresholds (Zi > 2.5 for module hubs, Pi > 0.62 for connectors, Zi > 2.5 and Pi > 0.62 for network hubs).

## 3 Results

### 3.1 Community structures of archaea, bacteria, and eukaryotes

**Figure 1:**
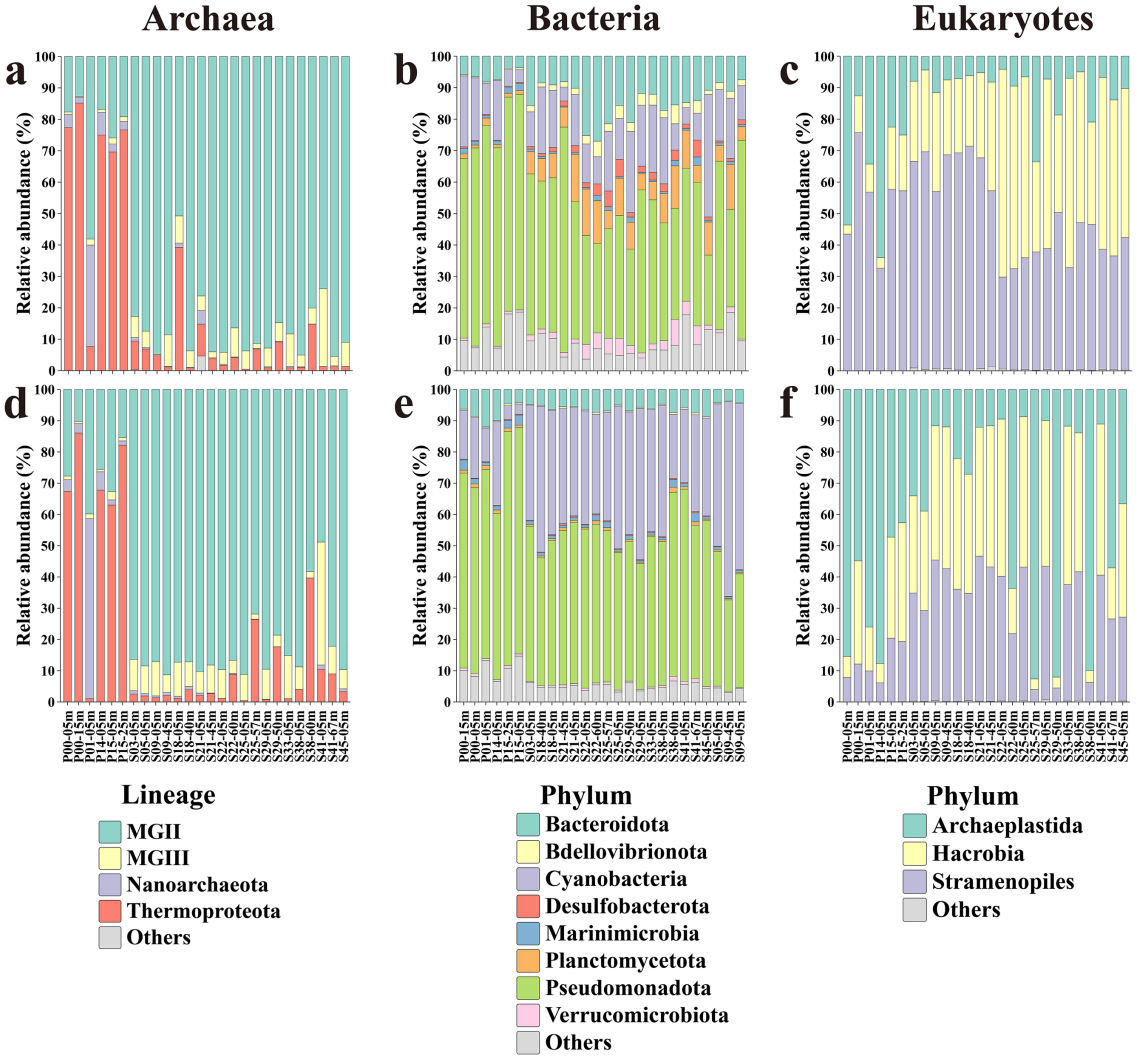
Phylum-level composition of bacterial and eukaryotic communities and dominant archaeal lineages. Stacked bar chart illustrating the relative abundance of **(a)** archaeal, **(b)** bacterial, and **(c)** eukaryotes phyla in PA communities. Stacked bar chart illustrating the relative abundance of **(d)** archaeal, **(e)** bacterial, and **(f)** eukaryotes in FL communities. For archaea, MGII and MGIII represent major archaeal lineages (Marine Group II and III) rather than formally described phyla and are shown separately due to their ecological importance.

After quality control, taxonomic filtering, and domain-specific rarefaction, 648 archaeal ASVs, 13,283 bacterial ASVs, and 2,543 eukaryotic ASVs were retained for community composition analyses. Microbial communities showed clear spatial and vertical variation across sampling sites (Figure 1). In archaeal communities, the community composition shifted markedly from the estuary to offshore waters. Thermoproteota dominated in the PRE (61–65%), followed by MGII (25%), while MGIII remained rare. In contrast, MGII was highly abundant in the NSCS (83%), and MGIII was more common in both PA and FL fractions compared with the PRE, suggesting a stronger adaptation of MGII/MGIII to oligotrophic offshore conditions. Bacterial communities were dominated by Proteobacteria, with Actinobacteriota enriched in the NSCS PA fraction and Cyanobacteria more abundant in the FL fraction, consistent with their photoautotrophic lifestyle. Among eukaryotes, Stramenopiles dominated PRE PA (58%), while Archaeplastida prevailed in PRE FL (66%). In NSCS PA, Hacrobia accounted for 39%, much higher than in the PRE (14%), implying regionally specific particle colonization patterns. These results indicated domain-specific community structure shaped by both environmental gradients and particle association.

The NMDS analysis indicated that two primary axes, extending from estuaries to offshore waters and from FL to PA habitats, collectively influence the communities. However, the strength and direction of these influences differ by domain (Supplementary Figure S2). Archaea did not display significant differences between FL and PA states (*p* > 0.05), but they did show a distinct regional separation (*p* < 0.01), indicating significant biogeographic differentiation rather than lifestyle-driven differentiation. Bacteria only exhibited segregation between FL and PA states in the NSCS (*p* < 0.01), whereas eukaryotes demonstrated pronounced differences in both regions (*p* < 0.01), indicating significant biogeographic differentiation. Overall, these results indicated that geographical factors dominate the differences in archaeal communities, while lifestyle differences shape the variations in bacterial and eukaryotic communities.

### 3.2 Environmental drivers of community structure

**Figure 2:**
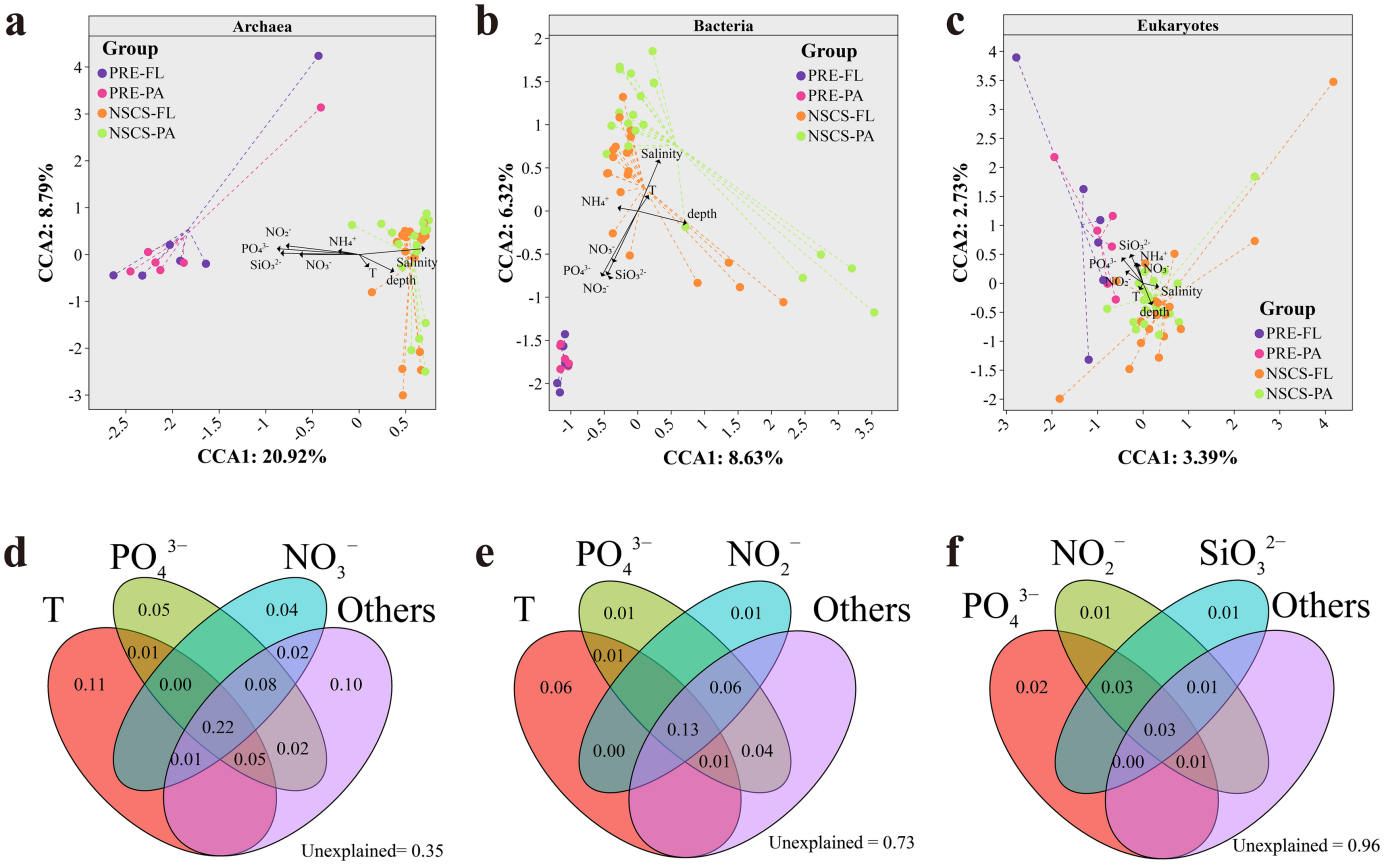
Relationship of nutrient environment and community. CCA chart showing the relationship nutrients with **(a)** archaeal, **(b)** bacterial, and **(c)** eukaryotic community. Colored lines link samples to their group centroids (PRP-FL/PA, NSCS-FL/PA), indicating within-group dispersion. Solid black arrows are environmental biplot vectors; arrow direction denotes increase of the variable and arrow length indicates the strength of correlation with the ordination. VPA chart showing the relative importance of nutrients to **(d)** archaeal, **(e)** bacterial, and **(f)** eukaryotic community.

Along the PRE to NSCS continuum, archaeal, bacterial, and eukaryotic communities separated mainly along the combined salinity and depth gradient, whereas vectors for nutrients such as nitrate (NO_3_^−^), nitrite (NO_2_^−^), ammonium (NH_4_^+^), phosphate (PO_4_^3−^), and silicate (SiO_3_^2−^) and temperature (T) indicated clear domain-specific sensitivities (Figures 2a–c). The environmental matrix included eight key hydrographic and nutrient variables, which showed no severe multicollinearity and were therefore all incorporated into the final CCA model. The first two canonical axes explained 29.7, 15.0, and 6.1% of the variance in archaeal, bacterial, and eukaryotic communities, respectively, indicating a progressively weaker coupling with the measured environmental variables from archaea to eukaryotes, with the strongest coupling in archaea and the weakest in eukaryotes. Pairs of FL and PA samples shifted roughly in parallel with the environmental vectors, consistent with a shared response to large-scale conditions, while PA samples were generally more dispersed, reflecting microhabitat heterogeneity of particles.

In the case of archaea, low-salinity estuarine samples and high-salinity, deeper offshore samples were distinctly separated along the first axis. The community structure correlated positively with salinity and depth, and inversely with several nutrients (Figures 2a,d). Variation partitioning attributed approximately 65% of archaeal variation to the measured environment, confirming a strong deterministic influence dominated by temperature at 0.11, phosphate at 0.05 and nitrate at 0.04. The interaction term was 0.22, indicating a synergistic effect between nutrients and physical factors. The FL and PA communities tended to converge offshore but remained distinct in the estuary, reflecting lifestyle-modulated environmental filtering.

In bacteria, both salinity and depth contributed to community differentiation and ammonium had an impact at NSCS (Figures 2b,e). The measured environment accounted for approximately 27% of the variation with modest unique effects from temperature (0.06), and minor contributions from nutrients (0.01). The substantial unexplained fraction (0.73) suggested that processes not captured by the measured environmental variables, such as dispersal limitation, stochasticity, or biotic interactions (e.g., grazing or viral lysis), may contribute to bacterial community variation. The significant FL-PA community differences observed in PRE are sufficient to support that bacteria adopt diverse lifestyles to achieve adaptation and survival when responding to variable marine environmental conditions.

For eukaryotes, separation in ordination space was weakest, and environmental vectors clustered near the origin (Figures 2c,f). Variation partitioning attributed only about 4% of the variation to the measured environment, suggesting dominance of unmeasured or stochastic processes. Unique and shared fractions were small, indicating that unquantified or stochastic processes, such as potential micro food web interactions, temporal lags, or particle size structure, likely play dominant roles.

Overall, the three domains exhibited an asymmetric response to environmental gradients. Archaea showed the strongest and most deterministic coupling with the environment, bacteria exhibited an intermediate response influenced by both environmental and stochastic factors, and eukaryotes were largely governed by stochastic or biotic interactions. Across all domains, PA communities were more spatially dispersed than FL counterparts, consistent with PA dispersal limitation and niche heterogeneity.

### 3.3 Assembly processes of archaea, bacteria, and eukaryotes

**Figure 3:**
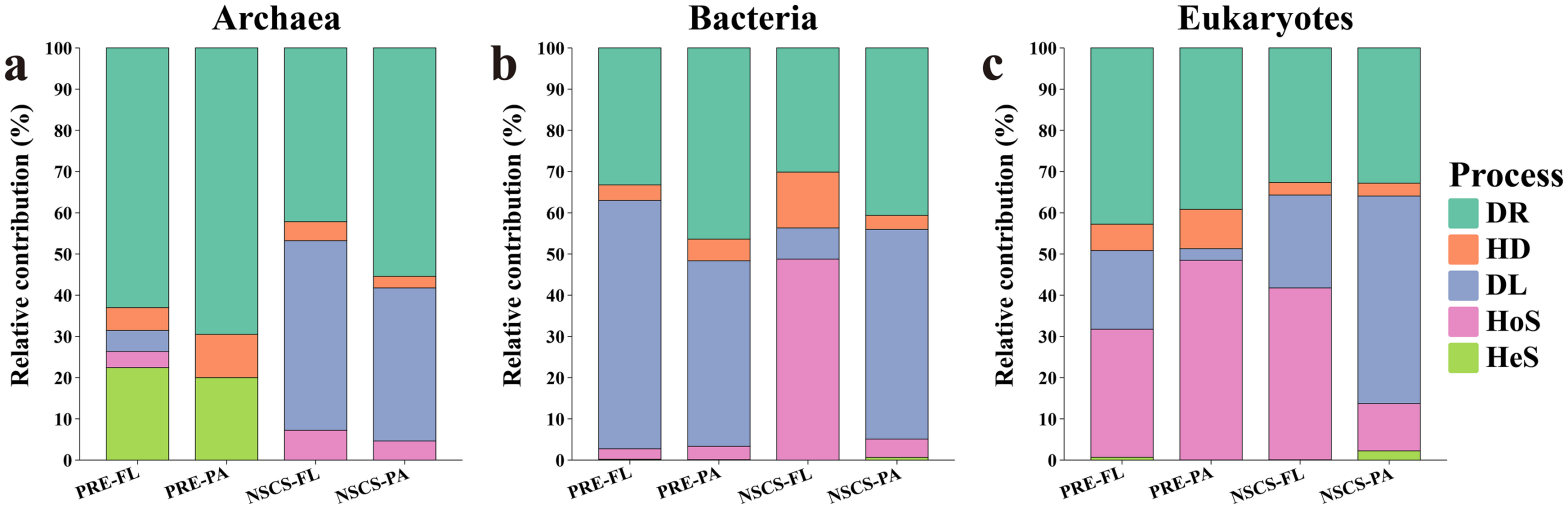
The processes of community assembly. Stacked bar showing the contribution of assembly processes in **(a)** Archaea, **(b)** bacteria, and **(c)** eukaryotes. DR, drift; DL, dispersal limitation; HD, homogenizing dispersal; HoS, homogeneous selection; HeS, heterogeneous selection.

For iCAMP analyses, low-abundance ASVs were filtered out to reduce stochastic noise, resulting in 183 archaeal, 931 bacterial, and 608 eukaryotic ASVs retained for downstream assembly inference. Null model partitioning revealed the assembly processes of three-domain microorganisms in different regions and lifestyles. In the archaeal community, the PRE group strongly favored drift, with heterogeneous selection providing a secondary influence (Figure 3a). In the NSCS, FL archaea appeared to be primarily influenced by dispersal limitation, while PA assemblages showed a mixed signature of drift and dispersal limitation, with only minor traces of selection or homogenizing dispersal. Overall, the archaeal evidence showed greater links to geographical factors than to environmental conditions.

In bacteria, patterns diverged according to lifestyle (Figure 3b). Whether it is PRE or NSCS, the PA community exhibited a combination of dispersal limitation and a relatively large proportion of drift. In PRE, the FL community primarily manifested as dispersal limitation, whereas in NSCS, homogeneous selection was the dominant process which showed greater environmental influence in offshore areas.

For eukaryotes, assembly exhibited a pronounced dependence on habitat. PRE-FL communities displayed a mix of drift and homogeneous selection, with drift being the dominant process. In contrast, PRE-PA communities were predominantly shaped by homogeneous selection. In the NSCS, FL eukaryotes predominantly experienced homogeneous selection with greater dispersal limitations compared to the estuary, whereas PA eukaryotes were primarily influenced by dispersal limitations (Figure 3c).

Overall, the assembly of communities were jointly structured by region and lifestyle, but their influence depends on specific ecological domains. Archaeal communities were more strongly influenced by drift in estuarine environments, whereas dispersal limitation dominated in offshore free-living assemblages. Bacteria showed that PA had dispersal limitation but NSCS-FL had homogeneous selection, suggesting the distribution patterns based on lifestyle. Eukaryotes showed the strongest habitat dependence because selection dominates in PA and offshore FL while dispersal limitation continues to affect PRE-FL and NSCS-PA.

### 3.4 Cross-domain interaction networks

**Figure 4:**
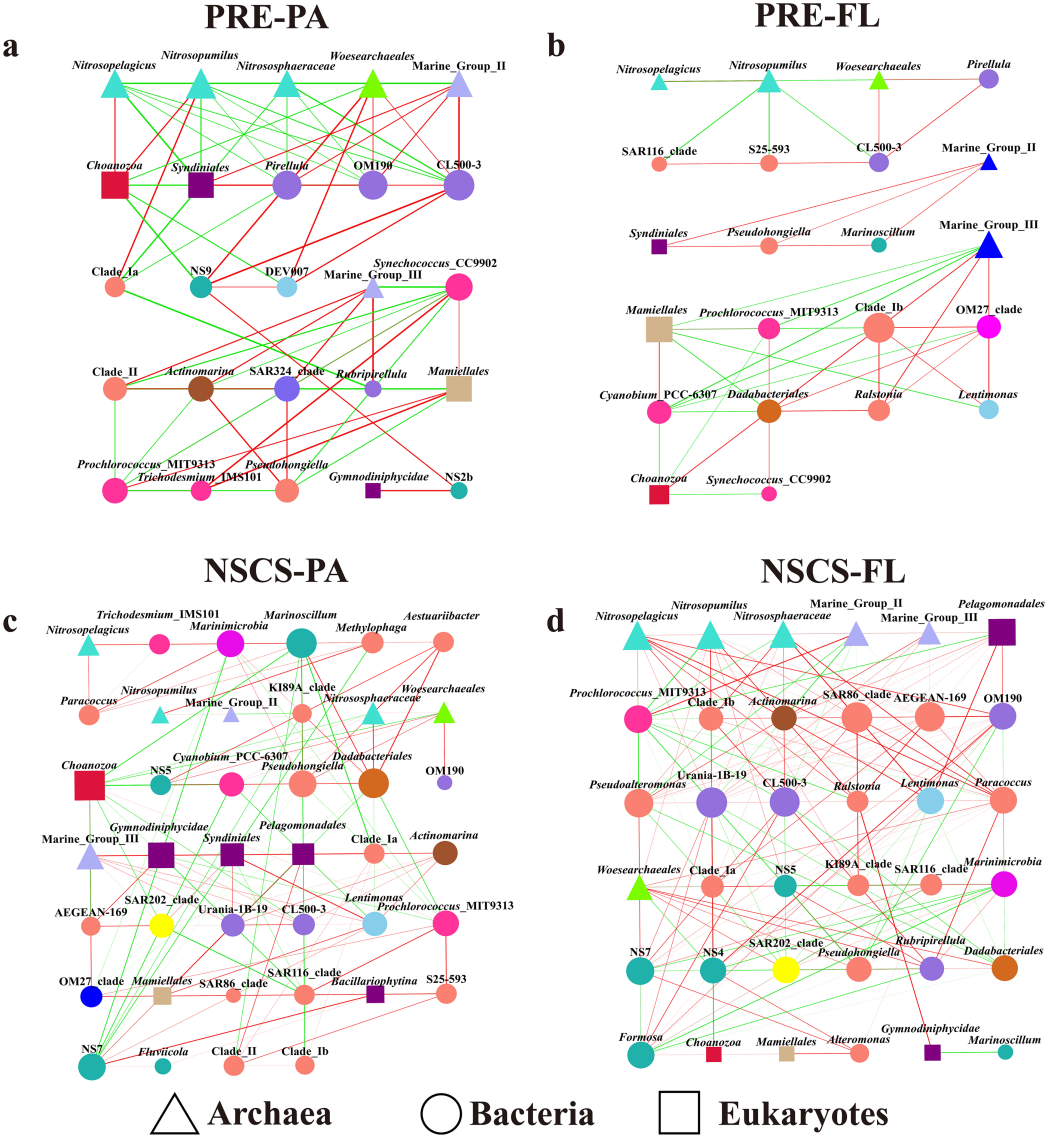
Interactions among archaea, bacteria, and eukaryotes. Triangles indicate archaea, circles represent bacteria, and squares represent eukaryotes. Subplot shows the **(a)** PA community in the PRP region, **(b)** FL community in the PRP region, **(c)** PA community in the NSCS region, and **(d)** FL community in the NSCS region. Regarding the edges, red lines indicate positive correlations, while green lines indicate negative correlations. Node colors distinguish different taxonomic groups at the phylum or class level.

Networks were constructed at the genus level using a stringent relative abundance threshold (≥1%), resulting in a limited number of dominant archaeal and eukaryotic nodes but a larger set of bacterial genera, thereby emphasizing robust and ecologically interpretable cross-domain interactions. Network topology changed systematically with both geographic region and lifestyle (Figure 4; Supplementary Table 2). The NSCS-FL network formed the densest web and contained the most cross-domain links, whereas PRE-FL network was comparatively sparse and weakly connected. Both PA networks were more modular, consistent with particles acting as discrete microhabitats that partition niches. Because the number of available samples differed between PRE and NSCS (6 vs. 20), network metrics related to connectivity should be interpreted in a relative rather than absolute sense. Nevertheless, the qualitative patterns reported here, including higher modularity in PA networks and greater cross-domain connectivity in NSCS networks, were consistent across regions and therefore unlikely to be driven solely by differences in sampling effort.

The PRE-PA network was dominated by archaeal bridge taxa, particularly AOA such as *Nitrosopelagicus* and *Nitrosopumilus*, which linked archaeal and eukaryotic modules (Figure 4a). Choanozoa and *Syndiniales* served as key eukaryotic mediators, showing positive associations with AOA but negative correlations with co-occurring bacteria. *Prochlorococcus* has a negative correlation with other bacteria, but a positive correlation with the eukaryotic group *Mamiellales*. *Syndiniales*, MGII, and *Pirellula* or OM190 formed positive three-domain co-occurrence modules, highlighting archaeal-bacterial-eukaryotic coupling on particles.

In contrast, the PRE-FL network displayed low connectivity and strong modular isolation (Figure 4b). MGIII developed unfavorable bonds with *Mamiellales* and *Prochlorococcus* but MGII built favorable connections with *Syndiniales* and *Marinoscillum*. The absence of dominant bridge taxa indicated weak cross-domain coupling in the highly dynamic estuarine water column.

The NSCS-PA networks showed increased structural complexity because they used eukaryote-based bridging connections (Figure 4c). The *Syndiniales* and *Pelagomonadales* groups served as vital connections between different domains because they formed beneficial relationships with MGIII and heterotrophic bacteria but showed negative interactions with various archaea and bacterial species. The *Choanozoa* clade presented wide-ranging negative relationships among its different groups, whereas MGII maintained relatively few connection points. The observed patterns demonstrated that offshore particles need eukaryotic mediators and heterotrophic bacteria to establish connections between different modules.

The NSCS-FL network formed a highly coupled structure, anchored by AOA (*Nitrosopelagicus*, *Nitrosopumilus*) and core heterotrophic clusters such as SAR86, SAR116, and AEGEAN-169 (Figure 4d). *Pelagomonadales* served as cross-domain connectors but *Mamiellales* and *Choanozoa* maintained limited negative connections between their groups. The *Prochlorococcus* MIT9313 strain established itself in the middle through its negative connections to heterotrophic clusters yet AOA maintained high betweenness centrality, indicating their bridging role between functional modules.

Overall, the research results showed that geographical position together with individual behaviors determine how different domains connect through their networks. The PRE-PA network needed AOA bridges while NSCS-PA network needed eukaryotic mediators to operate and NSCS-FL network depended on heterotrophs and AOA for their co-anchoring. The PRE-FL community showed the lowest connection strength because domain coupling remains weak when stochastic conditions become dominant. This progressive shift from archaeal to eukaryote-dominated bridging reflects an ecological transition from environmental filtering to functional integration along the PRE-NSCS continuum.

### 3.5 The core taxa in the community

The top 20 microbial taxa at the ASV level from the three domains were identified based on their cumulative relative abundance across all samples and subjected to phylogenetic and relative abundance analyses to determine the dominant lineages shaping community structure (Supplementary Table 3). The research showed that core taxa exist throughout all regions but domain groups showed distinct abundance patterns which indicated their success in different ecological environments from PRE to NSCS.

**Figure 5:**
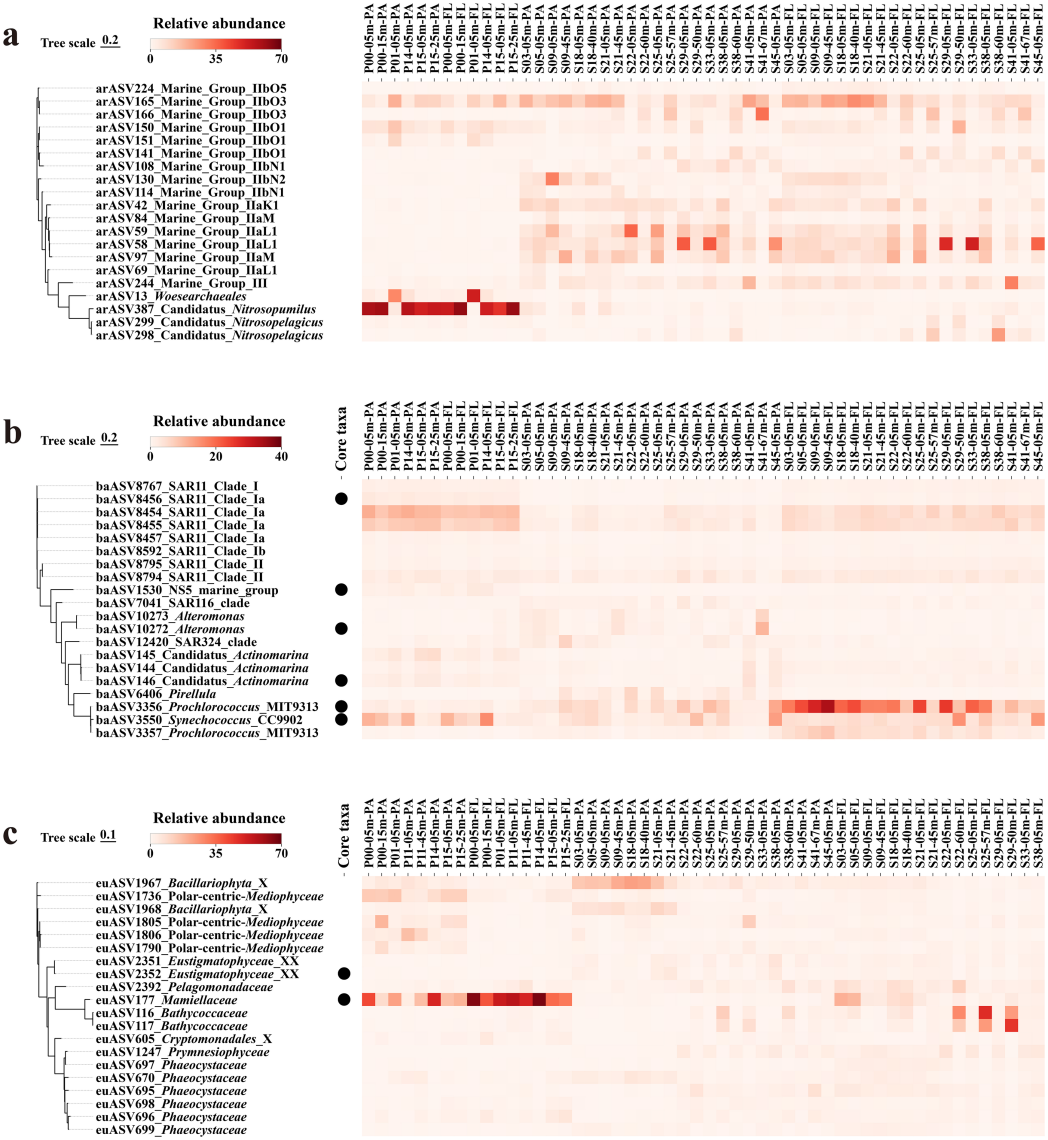
Phylogenetic tree and relative abundance of dominant lineages across the PRP–NSCS transect. The panels show **(a)** Archaea, **(b)** Bacteria, and **(c)** Eukaryotes. The left side presents phylogenetic trees of representative ASVs, and the right side shows heatmaps of relative abundance (%) across samples grouped by region (PRP versus NSCS) and lifestyle (FL versus PA).

Among the top 20 most abundant archaeal lineages, MGII and AOA (mainly *Ca. Nitrosopelagicus*) were most prominent (Figure 5a). Most subgroups of MGII, MGII-bO1, MGII-bN1, MGII-bN2, MGII-aK1, MGII-aM, and MGII-aL1, maintained low and stable levels across different sites, but their relative abundance remained higher in NSCS than in PRE because they thrive in offshore oligotrophic waters. Conversely, the PRE samples showed high *Ca. Nitrosopelagicus* levels which indicated AOA prefer to live in areas with elevated nutrient levels and particular redox gradient conditions.

The bacterial community consisted mainly of oligotrophs and phototrophs which included SAR11, *Prochlorococcus*, *Synechococcus*, *Alteromonas* (Supplementary Figures S3a–c). SAR11 was the most ubiquitous group, appearing in almost all samples, with Clade Ia showing a stable high proportion (Figure 5b). *Prochlorococcus* and *Synechococcus* were significantly enriched in NSCS-FL because they thrive best in environments with plenty of light and limited nutrient availability. The opportunistic copiotroph *Alteromonas* showed occasional peak occurrences because it responds to short periods of organic matter availability.

The eukaryotic assemblages exhibited significant spatial and temporal differences (Figure 5c). The *Bacillariophyta*, *Cryptomonadales*, and *Phaeocystaceae* occasionally bloomed at certain sites but *Mamiellaceae* established a permanent presence in the PRE which linked primary producers to microbial heterotrophs. Despite their low abundance, *Eustigmatophyceae* were consistently detected at multiple sites which showed they can thrive in various environmental conditions. The observed patterns indicated that eukaryotic microalgae generate local primary production and form particles which affect the habitats of bacteria and archaea.

Across the PRE-NSCS continuum, the three domains maintained broadly similar dominant taxonomic groups, but their relative abundances and spatial distributions varied markedly between regions. This pattern suggests that environmental constraints and lifestyle strategies shape geographic differentiation without completely restructuring the core taxonomic composition. The oligotrophic NSCS contains MGII and SAR11 as its dominant species but AOA and *Mamiellaceae* predominate in the nutrient-rich PRE. The three taxonomic groups function as operational bases which facilitate carbon-nitrogen coupling and cross-domain interactions between estuarine and offshore environments.

## 4 Discussion

**Regulation of community structure by geographic gradients and lifestyle**. Along the PRE to NSCS gradient, microorganisms in all three domains exhibit regional and lifestyle (FL/PA) distinctions. Our integrated analysis revealed that geographic gradients and particle partitioning jointly shape microbial community assembly and network connectivity, balancing stochastic and deterministic processes. Although all three domains respond to the PRE-NSCS gradient, the relative importance of deterministic versus stochastic processes differs markedly among archaea, bacteria, and eukaryotes, reflecting fundamental differences in their physiological traits, ecological niches, and modes of interaction with the environment. Along the PRE-NSCS gradient, community assembly and network connectivity reflect contrasting balances between stochastic and deterministic processes. In the dynamic, nutrient-rich PRE, stochastic influences are more prominent, resulting in compositionally variable and weakly connected networks, whereas in the oligotrophic NSCS, stronger environmental selection favors more stable and tightly coupled communities with coherent ecological roles. The environmental conditions of offshore waters differ from estuaries because they experience stable conditions and nutrient scarcity which creates strong environmental selection pressure (Stegen et al., 2012; Zhou and Ning, 2017; Gong et al., 2023).

Archaeal communities are predominantly governed by large-scale physicochemical gradients, notably salinity and depth, resulting in significant geographic segregation (Figure 2). This strong geographic structuring likely reflects the relatively narrow ecological niches and high physiological specialization of many archaeal taxa, particularly AOA, which are highly sensitive to large-scale physicochemical constraints such as salinity and nutrient concentrations. The observed pattern is consistent with previous research that demonstrated salinity and depth gradients control of archaeal distribution patterns in marine and estuarine environments (Xie et al., 2014; Simon et al., 2015). In the NSCS, AOA are detected in both PA and FL fractions, highlighting their potential involvement in nitrogen cycling under oligotrophic conditions. A large number of studies have reported the fundamental role of AOA in nitrogen cycling in oligotrophic marine environments. However, the role of AOA in nitrogen cycling in the particulate state is relatively scarce (Santoro et al., 2015; Liu et al., 2023). Bacterial communities exhibited a stronger response to local-scale heterogeneity, particularly PA microenvironments. This pattern is consistent with the high metabolic versatility, rapid growth rates, and functional redundancy of bacteria, which enable them to efficiently exploit organic-rich particles and respond rapidly to transient resource pulses.

In contrast, eukaryotic community assembly was more strongly influenced by trophic interactions and strategies. Their larger cell sizes, complex life cycles, and dependence on grazing, parasitism, or symbiosis make eukaryotes particularly sensitive to biotic interactions rather than solely to abiotic filtering. It should be noted, however, that for eukaryotes the observed FL-PA differentiation likely reflects not only lifestyle differences but also inherent size-related partitioning, as the serial filtration approach (2.7 μm and 0.22 μm) preferentially separates small pico- and nano-eukaryotes from larger micro-eukaryotes. Thus, the ordination patterns for eukaryotic communities likely integrate both lifestyle-associated habitat preferences and size-structured community composition, rather than representing a purely lifestyle-driven signal. Future studies combining multi-size fractionation, imaging-based approaches, or trait-resolved methods would help disentangle size versus lifestyle effects more explicitly. Our study suggested a hierarchical assembly framework with large-scale nutritional and hydrological conditions dictating the regional taxa pool, whereas particle microenvironments foster fine-scale niche differentiation.

**Cross-domain network connectivity, core taxa, and ecological stability shaped by lifestyle.** Particles serve as heterogeneous hotspots that reshape cross-domain interactions, enhancing microscale heterogeneity and cross-domain interactions as critical hubs for energy and nutrient exchange (Milke et al., 2022; Zhu et al., 2024). These contrasting network roles further reflect domain-specific ecological strategies, with archaea contributing primarily to core biogeochemical transformations, bacteria facilitating metabolic coupling, and eukaryotes acting as interaction mediators across trophic levels. PA networks exhibit higher modularity and complexity than FL counterparts, indicating enhanced niche differentiation and metabolic complementarity on particles. The core taxa of the ecosystem use different ecological approaches which work together to maintain ecosystem stability. In the PRE, PA AOA function as keystone taxa, bridging nitrification modules with heterotrophic clusters. In the marine environment, *Ca. Nitrosopelagicus* thrives in PRE, while MGII archaeal populations maintain stability in the NSCS (Santoro et al., 2015; Rinke et al., 2019). In contrast, NSCS PA networks rely on eukaryotic mediators (e.g., *Pelagomonadales* and *Syndiniales*) and heterotrophic bacteria for cross-module connectivity. The NSCS maintains autotrophic-heterotrophic relationships through its core bacterial species SAR11 and *Prochlorococcus* and *Synechococcus* but *Alteromonas* shows opportunistic growth patterns when the environment experiences occasional disturbances (Pedler et al., 2014; Bolaños et al., 2022).

FL networks in the NSCS form a tightly coupled backbone between AOA and heterotrophic bacteria, while PRE FL communities show minimal connectivity due to strong stochastic isolation. The nearshore areas contain eukaryotic communities that consist mainly of *Bacillariophyta* and *Mamiellaceae* which quickly adapt to changing nutrient levels. Collectively, these findings demonstrated that network architecture and stability are jointly determined by geographic context and lifestyle, with particles enhancing localized interactions and FL communities maintaining broader ecological stability (Wang et al., 2024; Lee et al., 2025). The ecosystem maintains its biogeochemical operations through a balanced mix of permanent taxa and adaptable taxa which adapt to changing environmental conditions. Importantly, these lifestyle-dependent patterns differentially affected the three domains, with particle association amplifying deterministic interactions for bacteria and eukaryotes, while archaeal assembly remained primarily constrained by broader physicochemical gradients.

**Synthesis and ecological implications.** We propose a conceptual framework for marine coastal systems, in which archaea are primarily structured by large-scale environmental filtering, bacteria by microhabitat-driven niche differentiation, and eukaryotes by trophic interactions and life-history constraints. The balance between deterministic and stochastic processes not only shapes community structure but also likely contributes to the stability and adaptability of marine microbial ecosystems. This synergy may influence microbial remineralization pathways, referring to the microbially mediated degradation and transformation of particulate and dissolved organic matter, as well as the partitioning between particulate and dissolved organic carbon export, thereby linking community assembly to biogeochemical feedbacks relevant for predicting marine carbon sequestration and material fluxes (Nguyen et al., 2022).

## 5 Summary and conclusion

Along the PRE-NSCS continuum, our analyses suggested that geographic filtering and particle partitioning jointly shape cross-domain microbiomes. Canonical ordinations indicated a decreasing tendency of community-environment coupling from archaea to bacteria to eukaryotes, with archaeal assemblages most closely aligned with salinity and depth, bacteria also responding to ammonium and phosphate, and eukaryotes showing weak linear associations. Variation partitioning further suggested that the measured environmental variables explained a higher proportion of variance in archaeal communities than in bacterial or eukaryotic communities, pointing to an asymmetric sensitivity to environmental forcing. Assembly analyses resolved patterns consistent with a regional contrast: in the dynamic, nutrient-rich PRE, signals of stochastic influences were more prevalent, whereas in the oligotrophic NSCS signals of homogeneous selection increased. These differences were reflected in network. On particles, PRE communities appeared to be bridged by AOA, potentially linking nitrification modules to heterotrophs. NSCS particle networks were more often mediated by eukaryotic connectors together with heterotrophic bacteria. In the FL fraction, NSCS tended to form a tightly coupled AOA and heterotroph backbone, whereas PRE-FL remained comparatively sparse, consistent with stronger stochastic isolation. MGII, SAR11, *Prochlorococcus*, *Synechococcus*, and *Mamiellaceae* might coincid with these patterns as core taxa and imply domain-specific niche partitioning. Taken together, we propose a working framework in which geography constrains regional taxa pools via physicochemical filtering, while lifestyle modulates microscale interaction rules and network stability. These findings are of great significance for predicting biogeochemical feedbacks and carbon-nitrogen coupling under changing ocean conditions.

## Data Availability

The datasets presented in this study can be found in online repositories. The names of the repository/repositories and accession number(s) can be found in the article/Supplementary material.

## Footnotes

1
https://odv.awi.de/
