# Supplementary material for: Particle partitioning and geography drive divergent microbial assembly and network connectivity in coastal South China Sea
Source: Front Microbiol. 2026 Jan 23;16:1738577. doi: 10.3389/fmicb.2025.1738577 (PMC12875984; doi:10.3389/fmicb.2025.1738577)
Supplement: Supplementary file 1 [file Table_1.DOCX]

Supplementary Material

# Supplementary Figures

#
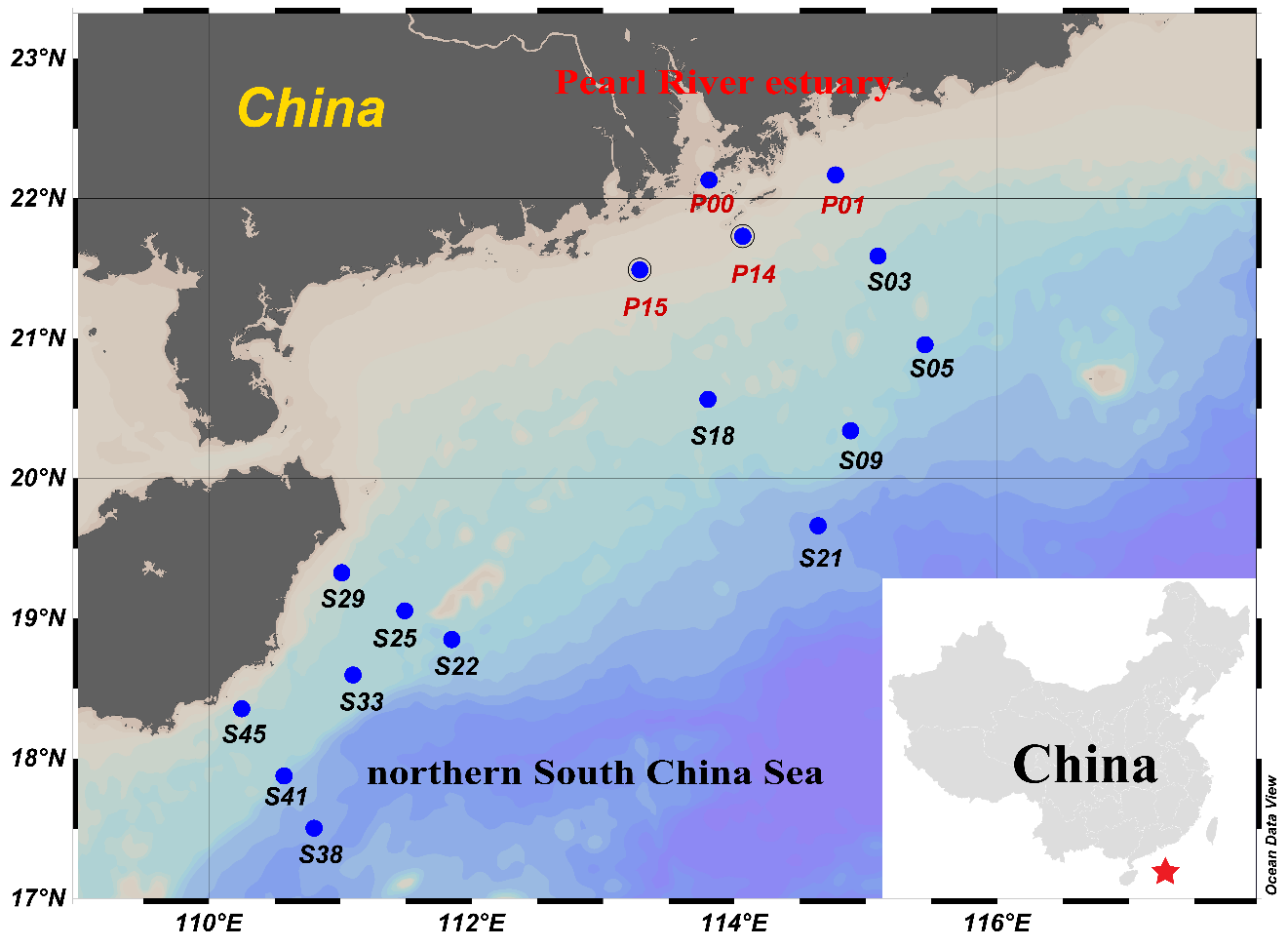


# Figure S1. Map illustrating the geographic sample locations. Sampling station locations are indicated by black and red words for NSCS and PRE, respectively.

#
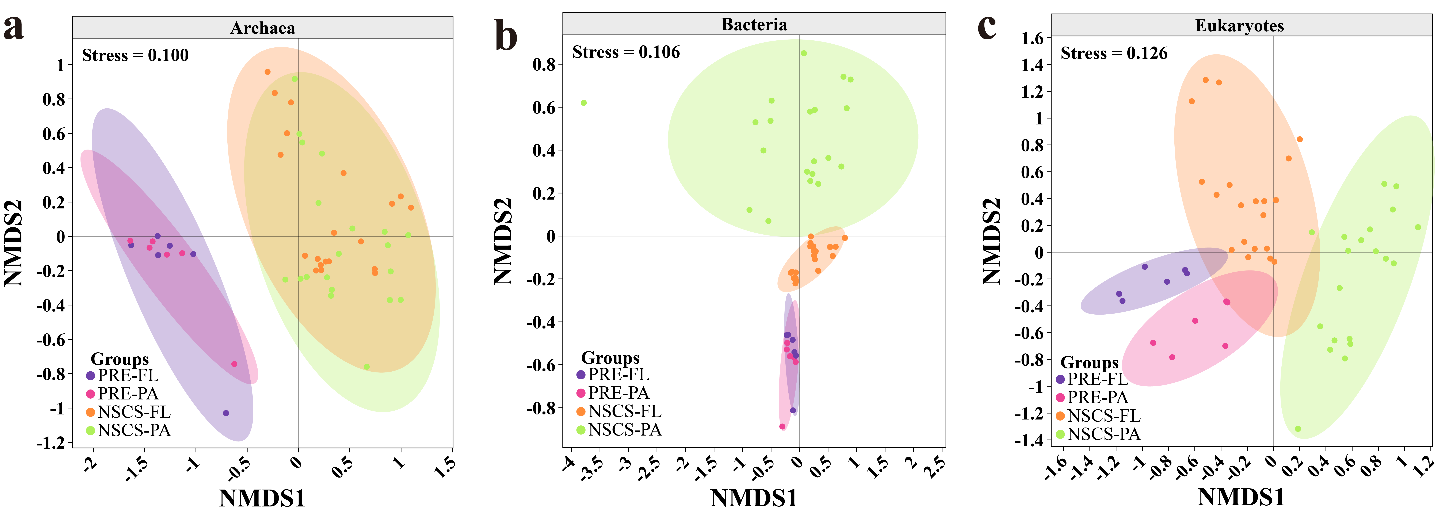


# Figure S2. Beta-diversity of microbiota. NMDS ordination chart illustrating the community structure of (a) archaeal, (b) bacterial and (c) eukaryotic community. Significance levels: p < 0.05, *p < 0.01, *p < 0.001.

#
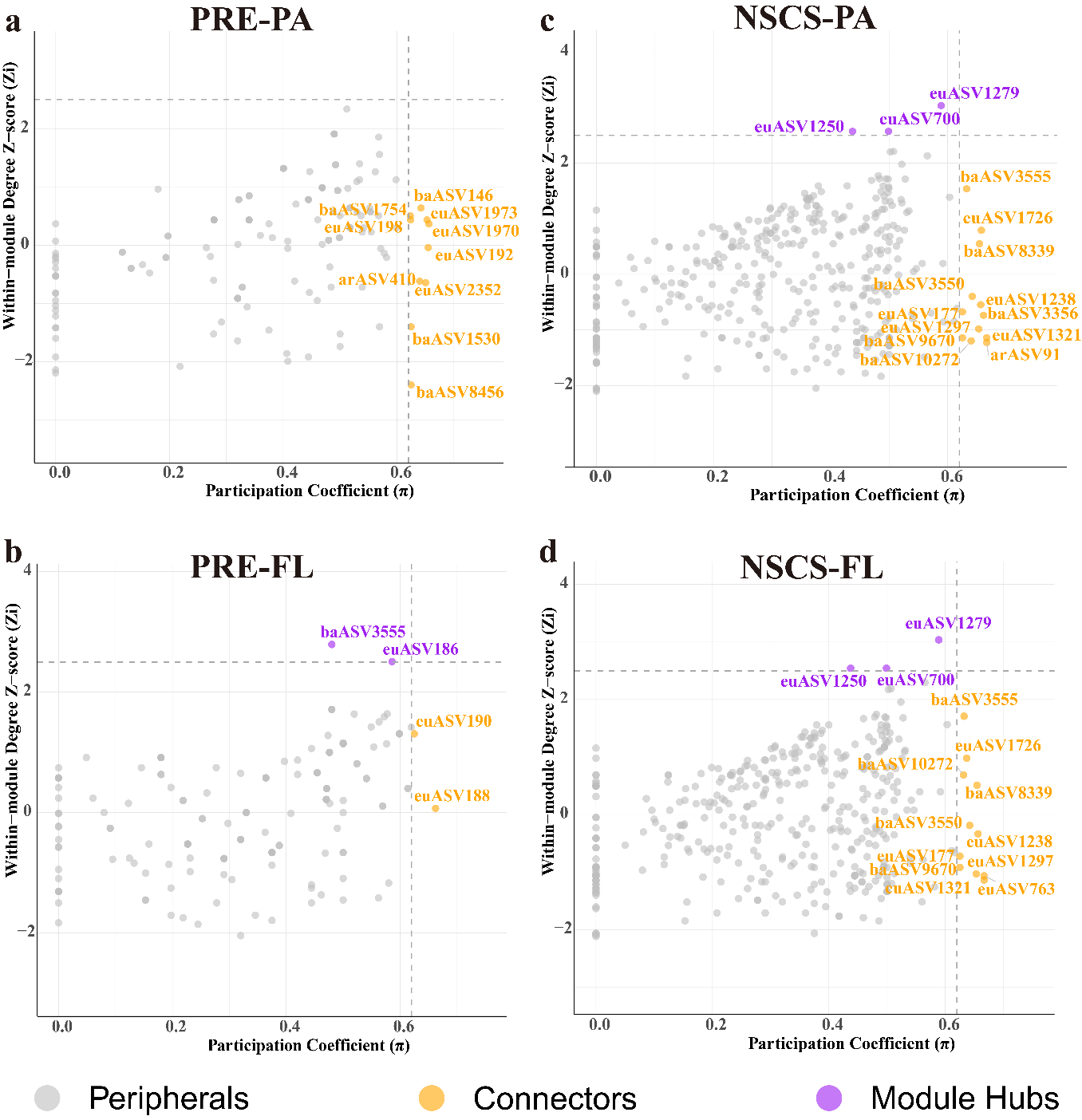


# Figure S3. Identification of keystone taxa. The plots show core species in (a) PRE-PA, (b) PRE-FL, (c) NSCS-PA, and (d) NSCS-FL, with a relative abundance >0.5% in at least one sample. Module hubs (Zi > 2.5), connectors (Pi > 0.62), and network hubs (Zi > 2.5 and Pi > 0.62) were considered as keystone taxa.
